# Local recurrence and metastatic disease in pheochromocytomas and sympathetic paragangliomas

**DOI:** 10.3389/fendo.2023.1279828

**Published:** 2023-12-07

**Authors:** Marta Araujo-Castro, Iñigo García Sanz, César Mínguez Ojeda, Felicia Hanzu, Mireia Mora, Almudena Vicente, Concepción Blanco Carrera, Paz de Miguel Novoa, María del Carmen López García, Cristina Lamas, Laura Manjón-Miguélez, María del Castillo Tous, Pablo Rodríguez de Vera, Rebeca Barahona San Millán, Mónica Recasens, Mariana Tomé Fernández-Ladreda, Nuria Valdés, Paola Gracia Gimeno, Cristina Robles Lazaro, Theodora Michalopoulou, Cristina Álvarez Escolá, Rogelio García Centeno, Verónica Barca-Tierno, Aura D. Herrera-Martínez, María Calatayud

**Affiliations:** ^1^ Endocrinology & Nutrition Department, Hospital Universitario Ramón y Cajal, Madrid, Spain; ^2^ Instituto de Investigación Biomédica Ramón y Cajal (IRYCIS), Madrid, Spain; ^3^ General & Digestive Surgery Department, Hospital Universitario de La Princesa, Madrid, Spain; ^4^ Urology Department, Hospital Universitario Ramón y Cajal, Madrid, Spain; ^5^ Endocrinology & Nutrition Department, Hospital Clinic, Barcelona, Spain; ^6^ Endocrinology & Nutrition Department, Hospital Universitario de Toledo, Toledo, Spain; ^7^ Endocrinology & Nutrition Department, Hospital Universitario Príncipe de Asturias, Madrid, Spain; ^8^ Endocrinology & Nutrition Department, Hospital Clínico San Carlos, Madrid, Spain; ^9^ Endocrinology & Nutrition Department, Hospital Universitario de Albacete, Albacete, Spain; ^10^ Endocrinology & Nutrition Department, Hospital Universitario Central de Asturias, Oviedo, Spain & Instituto de Investigación Sanitaria del Principado de Asturias (ISPA), Oviedo, Spain; ^11^ Endocrinology & Nutrition Department, Hospital Universitario Virgen de la Macarena, Sevilla, Spain; ^12^ Endocrinology & Nutrition Department, Institut Català de la Salut Girona, Girona, Spain; ^13^ Endocrinology & Nutrition Department, Hospital Universitario de Puerto Real, Cádiz, Spain; ^14^ Endocrinology & Nutrition Department, Hospital Universitario Cruces, Biobizkaia, Bizkaia, Spain; ^15^ Endocrinology & Nutrition Department, Hospital Royo Villanueva, Zaragoza, Spain; ^16^ Endocrinology & Nutrition Department, Hospital Universitario de Salamanca, Salamanca, Spain; ^17^ Department of Endocrinology and Nutrition, Joan XXIII University Hospital, Tarragona, Spain; ^18^ Endocrinology & Nutrition Department, Hospital Universitario La Paz, Madrid, Spain; ^19^ Endocrinology & Nutrition Department, Hospital Universitario Gregorio Marañón, Madrid, Spain; ^20^ Genetic Department, Hospital Universitario Ramón y Cajal, Madrid, Spain; ^21^ Endocrinology & Nutrition Department, Hospital Reina Sofía, Córdoba, Spain; ^22^ Endocrinology & Nutrition Department, Hospital Universitario Doce de Octubre, Madrid, Spain

**Keywords:** adrenal tumor, SDHB gene, recurrent disease, metastatic PPGL, catecholamines

## Abstract

**Purpose:**

To evaluate the rate of recurrence among patients with pheochromocytomas and sympathetic paragangliomas (PGLs; together PPGLs) and to identify predictors of recurrence (local recurrence and/or metastatic disease).

**Methods:**

This retrospective multicenter study included information of 303 patients with PPGLs in follow-up in 19 Spanish tertiary hospitals. Recurrent disease was defined by the development of local recurrence and/or metastatic disease after initial complete surgical resection.

**Results:**

A total of 303 patients with PPGLs that underwent 311 resections were included (288 pheochromocytomas and 15 sympathetic PGLs). After a median follow-up of 4.8 years (range 1-19), 24 patients (7.9%) had recurrent disease (3 local recurrence, 17 metastatic disease and 4 local recurrence followed by metastatic disease). The median time from the diagnosis of the PPGL to the recurrence was of 11.2 months (range 0.5-174) and recurrent disease cases distributed uniformly during the follow-up period. The presence of a pathogenic variant in *SDHB* gene (hazard ratio [HR] 13.3, 95% CI 4.20-41.92), higher urinary normetanephrine levels (HR 1.02 per each increase in standard deviation, 95% CI 1.01-1.03) and a larger tumor size (HR 1.01 per each increase in mm, 95% CI 1.00-1.02) were independently associated with disease recurrence.

**Conclusion:**

The recurrence of PPGLs occurred more frequently in patients with *SDHB* mutations, with larger tumors and with higher urinary normetanephrine levels. Since PPGL recurrence may occur at any time after the initial PPGL diagnosis is performed, we recommend performing a strict follow-up in all patients with PPGLs, especially in those patients with a higher risk of recurrent disease.

## 1 Introduction

Pheochromocytomas and paragangliomas (PGLs) −together PPGLs− are neuroendocrine tumors derived from chromaffin cells of the adrenal medulla or extra-adrenal paraganglionic tissue, respectively (1). They are considered rare tumors, occurring in about 0.05% to 0.1% of patients with sustained hypertension. It is estimated that the joint annual incidence of PPGL is of 2–8 cases per million inhabitants (1). Although they are rare tumors, they are considered one of the most frequent inherited tumors since about one tumor in four are linked to a genetic disease (2). The most common hereditary syndromes are those associated with pathogenic variants in the different subunits of *SDH* (15–20%), in the *VHL* gene (9%), in the *RET* proto-oncogene (5%) and in the *NF1* gene (2%). Currently, targeted Next Generation Sequencing (NGS) is the recommended approach to enable the testing of all relevant genes potentially associated with PPGL development in a single panel (3). The characterization of the genetic status of PPGL is of paramount importance given the well-known genotype-phenotype correlation in these tumors. This correlation includes associated biochemical profile, tumor location, malignant potential, and overall prognosis. In addition, genetic identification provides valuable information for establishing a treatment plan and procures the rational for an appropriate guidance for follow-up surveillance (4).

PPGLs are usually curable with the removal of the catecholamine secreting tumor. However, both pheochromocytomas and PGLs may recur as a benign or malignant tumor. In this sense, it is estimated that about 5% to 20% of PPGLs exhibit recurrence, and it can occur even after several decades after primary tumor resection (5–8). This fact justifies the need of a long-term follow-up for all patients with PPGLs who have undergone surgery (9). Surgery after local recurrence of PPGLs represents a major technical challenge and the only curative option for these patients. Although laparoscopic resection is possible in selected cases, it may be limited by the presence of multiple associated neoplasms and the impossibility of lymph node clearance (7). Another, even more challenging situation, is the development of metastatic disease, since therapeutic options for metastatic disease are limited; thus the management of all of these cases should be carried out by a multidisciplinary reference team (10). In this context, some factors, including genetic status, tumor size and location, among others, have been associated to a higher likelihood of metastatic PPGLs development (11–17). In this regard, some studies found that plasma methoxytyramine is the most accurate biomarker for discriminating patients with and without metastases, with a plasma methoxytyramine 4.7-fold higher in patients with than without metastases (11). Other authors describe larger increases of norepinephrine in malignant than in benign disease (14). A higher malignant risk associated with tumors due to mutations of *SDHB* gene or arising from extra-adrenal locations has been reported by several series (12, 15). Most studies also agree that there is an association between tumor size and the risk of malignancy in PPGLs (15–17). Other authors identified as risk factors of metastatic disease, an early onset postoperative hypertension, higher plasma or urine metadrenaline and the expression of the 3 angiogenesis related genes *VEGF*, *COX-2* and *MVD* (17). Thus, in general, there is no clear consensus on which are the risk factors for metastatic disease in PPGLs. In addition, the available data on the natural history of pheochromocytomas and PGLs after radical surgery are heterogeneous and discordant. Moreover, most of these studies are unicentric and include a limited number of cases. Thus, considering this background, the aim of our study was to find clinical predictors of recurrence (including local recurrence and metastatic disease) in patients with pheochromocytomas and sympathetic PGLs that underwent radical surgery.

## 2 Methods

### 2.1 Study design

**Figure 1:**
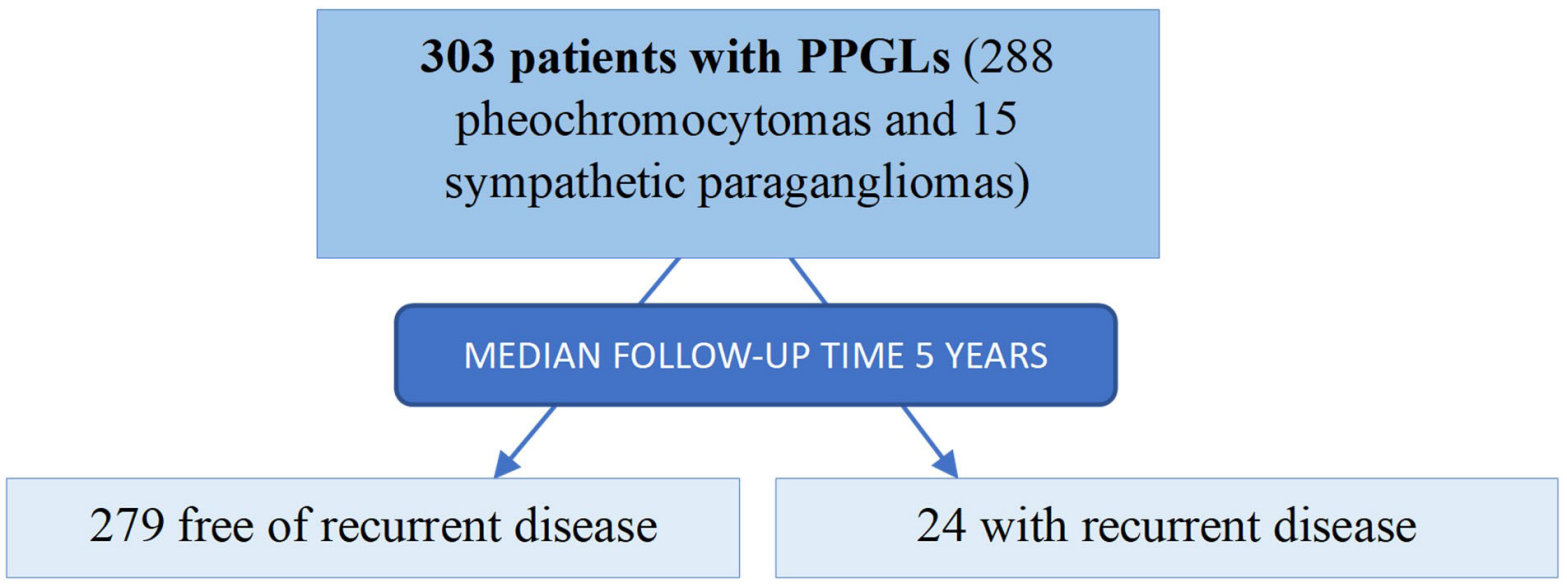
Study population.

A Spanish multicentric retrospective study of patients who underwent surgical resection of a PPGL between 1998 and 2022 in 19 tertiary hospitals was carried out. As we have previously described (18), the following criteria should be met to enter in the PHEO-PARA risk study: i) an age at diagnosis of the PPGL older than 17 years old; ii) histological diagnostic confirmation of PPGL, iii) available clinical, biochemical, and radiological information at the diagnosis of the PPGL and during follow-up and iv) absence of evidence of metastatic disease at the time of the diagnosis. Among all patients, 303 patients with PPGLs who underwent to 311 resections met the inclusion criteria and were included (288 pheochromocytomas and 15 sympathetic PGLs) (**Figure 1**).

The Ethics Committee of Hospital Universitario Ramón y Cajal has reviewed and approved the study on 22^nd^ of April 2021, ACTA 411.

PPGL: pheochromocytomas and sympathetic paragangliomas. After a median follow-up of 4.8 years (range 1 to 19), 24 patients (7.9%) had recurrent disease (3 local recurrence, 17 metastatic disease and 4 local recurrence followed by metastatic disease).

### 2.2 Clinical evaluation and definitions

The diagnosis of PPGL was based on the recommendations of the current clinical guidelines (9, 19). Catecholamine hypersecretion was assessed by the determination of plasma-free metanephrines, 24-h urinary fractionated metanephrines and/or 24-h urinary catecholamines. Considering that different normal ranges were applied for these determinations in the different local laboratories, the number of times (standard deviations: SD) above the upper limit of normal for each value was calculated and used for the analysis.

Hereditary PPGL diagnosis was based on the presence of a pathogenic germline variant in known susceptibility genes. As we have previously described (18), in all patients with a negative genetic study, at least the following genes have been sequenced: *NF1, RET, VHL, SDHA, SDHB, SDHC*, and *SDHD.* In addition, most of the centers tested also other genes, including *SDHAF2, SDHAF1, MAX, HIF1A, HIF2A, TMEM127, HRAS, KRAS, GOT2, FH, MDH2, SLC25A11, DNMT3A, DLST, MERTK, IDH1, IDH2, CSED1, EGLN1, EGLN2, BRAF, MET, FGFR1, KIF1B,CDKN1B, MEN1, PTEN, H3F3a, ATRX*, and the promoter region of *TER.* Positive genetic study was based on the demonstration of a pathogenic variant in at least one of these genes; those cases with variants of uncertain significance (VUS) were excluded. Cardiovascular disease was defined as the presence of ischemic and/or hypertensive heart disease, heart failure, cardiac arrhythmias and/or valvular disease. Obesity was defined as a body mass index ≥ 30 kg/m^2^ and diabetes definition was based on the last American Diabetes Association (ADA) recommendations (20). As we have previously reported (21), hypertensive PPGL was defined when systolic blood pressure was > 140 mmHg and/or diastolic blood pressure > 90 mmHg before surgery, or the patient was under medical treatment with antihypertensive drugs.

Recurrent disease was defined as the development of a local and/or metastatic disease during follow-up after the confirmation of surgical cure. Local recurrence was diagnosed when a local relapse occurred; and we considered metastatic PPGLs when recurrence occurred at sites where chromaffin tissue is normally absent. Follow-up period was defined as the time between the date of the PPGL diagnosis to the last available follow-up visit in the Endocrinology Department in patients with non-recurrent disease, and between the date of the PPGL diagnosis to the date of the diagnosis of recurrent disease in patients with recurrent PPGL. All patients were followed-up annually with hormonal values and with CT/MRI associated with nuclear medicine imaging if there were suspicions of tumor recurrence.

### 2.3 Statistical analysis

The statistical analysis was performed using STATA.15 (StataCorp LLC, College Station, Texas, USA). Continuous variables were described as means ± SD for normally distributed data or medians and interquartile ranges for non-normal distributions and compared using two-tailed t test. Categorical variables were expressed as percentage and absolute numbers and were compared using the chi2 test. To describe the timing of recurrence, the cumulative incidence was estimated using the Kaplan−Meier method. Statistical significance (p < 0.05) of differences in the cumulative incidence of recurrence between groups was tested using the log-rank test for homogeneity. A univariant Cox proportional hazard model was employed to estimate the crude hazard ratios (HRs) and the multivariant Cox proportional hazard model for the estimation of multivariable-adjusted HRs with 95% confidence intervals (CIs) and to evaluate possible predictors of recurrence.

## 3 Results

### 3.1 Baseline characteristics

A total of 303 patients with PPGLs that underwent 311 resections were included (288 pheochromocytomas and 15 sympathetic PGLs). Open surgery was performed in 41 PPGLs (including thoracotomy in 2 sympathetic PGL) and in the rest of the cases laparoscopic surgery was performed. Hereditary PPGL was confirmed by genetic analysis in 93 out of the 265 patients (35.1%) with available genetic results (in the remaining 38 patients, genetic study was not performed, or results were still pending). The most common pathogenic variant was in the *RET* gene causing MEN2A syndrome (n=45), followed by in the *NF1* gene (n=19), in the *SDHB* gene (n=14) and in the *VHL* gene (n=6). The pathogenic variant on these genes is not known in all cases, but for the RET pathogenic variant this information was available in 14 cases, being the mutation in exon 11 at codon 634 (p.Cys634Phe; TGC-TTC; n=8) and at codon 618 (p.Cys618Arg;:TGC>CGC; n=5) the most common pathogenic variants. One patient had a mutation at codon 634 (p.Cys634Tyr; TGC>TAC), and the information was lacking for the remaining patients. Personal and clinical characteristics of the patients at the time of the diagnosis are shown in **Table 1**.

**Table 1:** Baseline patient´s characteristics.

Variable	Patients (n=303)
Female sex	51.8% (n=157)
Age (years)	52 (range 18-81)
Hereditary PPGL [n=265]	35.1% (n=93)
Hypertension	72.3% (n=219)
Systolic blood pressure at diagnosis (mmHg)	133 (range 96-220)
Diastolic blood pressure at diagnosis (mmHg)	80 (range 50-135)
Diabetes	26.4% (n=80)
Fasting plasma glucose levels (mg/dL)	101 (range 70-283)
Obesity	16.2% (n=49)
Body mass index, kg/m2 [n=272]	25.7 (range 17.6-43.9)
Cardiovascular disease	13.2% (n=40)
Cerebrovascular disease [n=301]	4.7% (n=14)
Smoker [n=265]	25.3% (n=67)
Glomerular filtration rate (ml/min/1.73m^2^)	85 (range 38-165)
Catecholamine phenotype [n=223]	32.7% noradrenergic; 17.9% adrenergic; 48.4% mixed and 1% dopaminergic
Urine metanephrine (SD) [n=175]	1.4 (range 0-85)
Urine normetanephrine (SD) [n=153]	2.4 (range 0.1-70)
Urine epinephrine (SD) [n=203]	1.4 (range 0-42)
Urine norepinephrine (SD) [n=212]	1.6 (range 0.1-46.5)
Urine dopamine (SD) [n=164]	0.5 (range 0-8.4)
Plasmatic metanephrine (SD) [n=68]	1.5 (range 0-31.6)
Plasmatic normetanephrine (SD) [n=67]	3.4 (range 0.3-35.9)
Tumor size (mm) [n=292]	42 (range 10-136)
Tumor >40 mm [n=292]	51.0% (n=149)
Bilateral tumor	4.6% (n=14)

PPGL, pheochromocytomas and sympathetic paragangliomas; SD, standard deviations. Normal range for systolic blood pressure at diagnosis: <140 mmHg; for diastolic blood pressure: <90 mmHg; for fasting plasma glucose: <100 mg/dL; for glomerular filtration rate: >90 ml/min/1.73m^2^. *For variables with missing data, the number of patients with available data is described in brackets.

### 3.2 Recurrent disease and survival analysis

**Figure 2:**
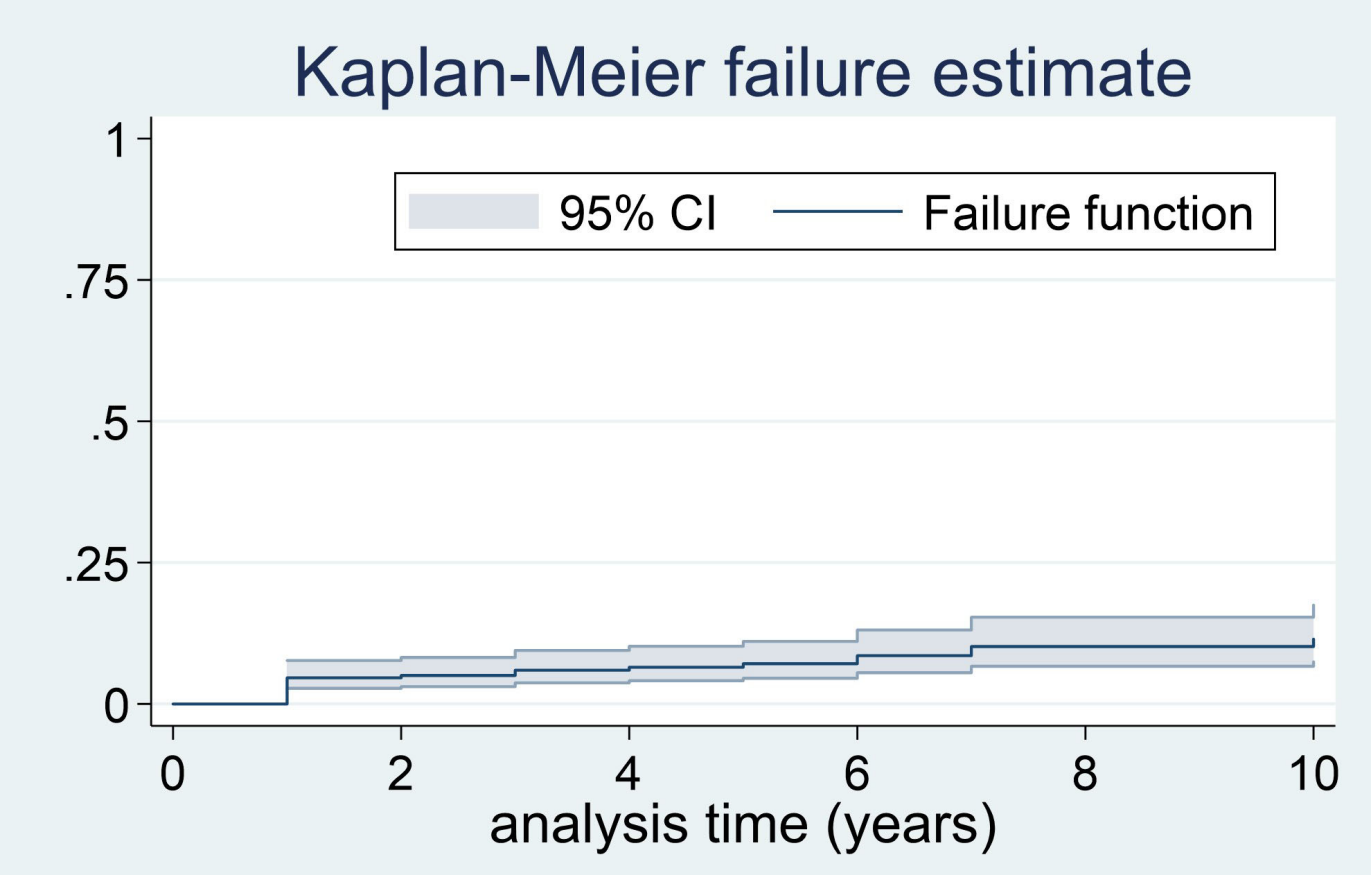
Failure curve for recurrent disease in PPGL.

After a median follow-up of 4.8 years (range 1 to 19), 24 patients (7.9%) had recurrent disease (3 local recurrence, 17 metastatic disease and 4 local recurrence followed by metastatic disease). The most common site of metastasis was the bone (n=9) and the lymph nodes (n=8), followed by the liver (n=5), lungs (n=5), retroperitoneum (n=4), peritoneum (n=2) and neck (n=1); 9 patients having metastasis in two or more sites. The median time from the diagnosis of the PPGL to the diagnosis of the recurrent disease was of 11.2 months (range 0.5 to 174), and the cases of recurrent disease distributed uniformly during the follow-up period (**Figure 2**). However, the higher cumulative incidence of new cases of recurrence was observed in the period of 0 to 2 years of follow-up (hazard function of 0.03, 95% CI 0.01 to 0.04) (**Table 2**). In addition, the overall follow-up time was longer in those patients who had recurrence compared with patients free of recurrence (8.1 ± 5.68 vs. 6.0 ± 4.63 years, P=0.040).

**Table 2:** Cumulative incidence of recurrent disease by intervals (years).

Interval (years)	Patients in follow-up	Cumulative incidence	Hazard	95% Confident interval
0-2	303	0.051	0.026	0.013-0.040
2-4	229	0.065	0.007	0.000-0.016
4-6	177	0.077	0.007	0.000-0.016
6-8	131	0.110	0.018	0.000-0.035
8-10	93	0.110	0.000	Not calculable
10-12	68	0.135	0.015	0.000-0.044

The cases of recurrence of the PPGL occurred progressively during the follow-up period, even after 10 years of follow-up. However, the higher risk of recurrence was observed in the period of 0 to 2 years (hazard: 0.026; 95% CI 0.013-0.040).

**Figure 3:**
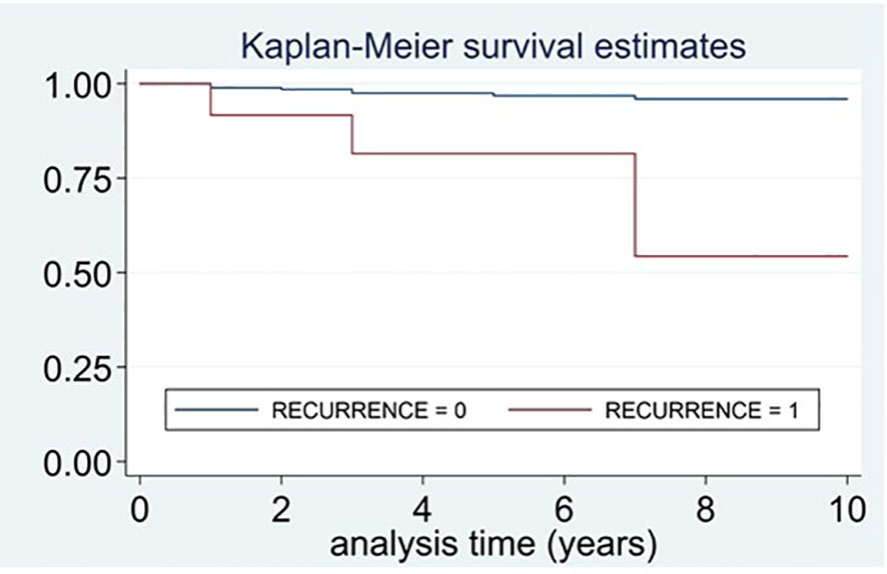
Survival curves of patients who died and had recurrent disease vs. patients who died and did not have recurrence.

A total of 12 patients died during follow-up (18.2% of the patients with recurrent disease vs. 2.9% of benign PPGLs, P<0.001). The survival time was significantly lower in those patients with recurrent disease in comparison with those without recurrence (Log-rank test for equality of survivor functions, *X* ^2^ 18.1, P<0.0001) (**Figure 3**).

The survival time was lower in those patients with recurrent disease than in those without recurrence (Log-rank test for equality of survivor functions, X2 18.1, P<0.0001).

### 3.3 Predictors of recurrent disease

**Figure 4:**
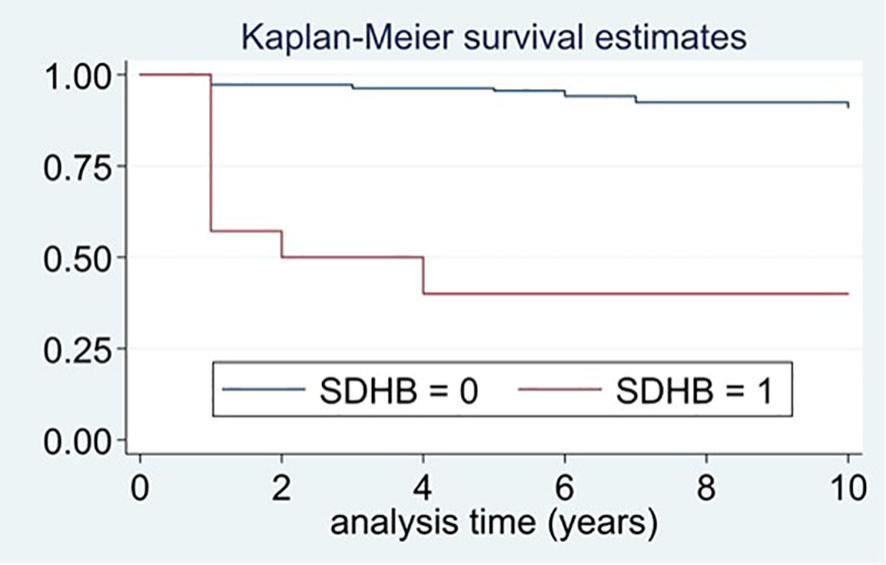
Differences in time free of recurrence between patients with and without pathogenic variants in *SDHB*.

In the univariant analysis, a hereditary PPGL, harboring a *SDHB* pathogenic variant, a higher excretion of urine normetanephrine and lower of urine epinephrine, a larger tumor size and having a sympathetic PGL were identified as predictors of recurrent disease (**Table 3**). *SDHB* mutation was the strongest predictive factor of recurrent disease. Moreover, patients with *SDHB* who had recurrence, developed the recurrence earlier than patients with recurrence without *SDHB* pathogenic variants (1.2 ± 1.67 vs. 3.8 ± 3.95 years, P=0.034). The number of recurrences was significantly higher in *SDHB* mutated PPGLs than in those without the mutation (events expected 23.1% vs. 0.9%, log-rank test; *X2* 63.0, P<0.0001). The median time free of recurrence was significantly higher in PPGL without pathogenic variants in SDHB than in those carrying the pathogenic variants (**Figure 4**). In fact, the association of recurrent disease and hereditary PPGL disappeared after adjusting by *SDHB* mutational status (adjusted HR 1.29 [0.46-3.66]). Similarly, the association between sympathetic PGL and risk of recurrence disappeared after adjusting by *SDHB* mutational status (adjusted HR 2.64 [0.43-16.46]). The variables that were independently associated with recurrence (in the multivariant analysis) were the presence of *SDHB* pathogenic variant (HR 13.3, 95% CI 4.20-41.92), higher levels of urinary normetanephrine (HR 1.02 per each increase in standard deviation, 95% CI 1.01-1.03) and a larger tumor size (HR 1.01 per each increase in mm, 95% CI 1.00-1.02). In addition, those patients operated by an open approach had a four-fold higher risk of recurrence than those operated laparoscopically (HR 3.62, 95% CI 1.58-8.29). These differences continued being statistically significant after adjusting by tumor size (HR 3.15, 95% CI 1.34-7.41), but disappeared after adjusting by *SDHB* mutational status (adjusted HR 2.01, 95% CI 0.81-4.98).

**Table 3:** Predictors of recurrent disease in PPGLs.

Variable	Recurrent disease (n=24)	Free of recurrent disease (n=279)	Hazard ratio [95% CI], p value
Male sex	62.5% (n=15)	47.0% (n=131)	1.87 [0.82-4.27], 0.131
Age	46.8 ± 13.52	51.6 ± 16.15	0.99* [0.96-1.01], 0.244
Age < 35 years	16.7% (n=4)	17.2% (n=48)	0.89 [0.30.2.59], 0.824
Hereditary PPGL [n=265]	60.9% (n=14/23)	32.6% (n=79/249)	2.72 [1.17-6.31], 0.018†
*SDHB* mutation	33.3% (n=8/24)	2.2% (n=6/279)	15.46 [6.45-36.80], <0.0001†
Hypertension	83.3% (n=20)	71.3% (n=199)	2.21 [0.75-6.47], 0.116
Diabetes	30.4% (n=7)	26.2% (n=73)	1.56 [0.64-3.79], 0.346
Obesity	12.5% (n=3)	16.5% (n=46)	0.75 [0.22-2.51], 0.628
Cardiovascular disease	4.2% (n=1)	14.0% (n=39)	0.32 [0.04-2.39], 0.184
Cerebrovascular disease [n=301]	4.2% (n=1)	4.7 (n=13)	0.79 [0.11-5.85], 0.811
Smoker [n=265]	17.4% (n=4)	26.1% (n=63)	0.61 [0.21-1.80], 0.346
Systolic blood pressure at diagnosis	135.9 ± 28.48	138.2 ± 25.57	1.00* [0.98-1.02], 0.754
Diastolic blood pressure at diagnosis	80.5 ± 14.59	82.0 ± 16.22	1.00* [0.97-1.02], 0.741
Secretory phenotype [n=223] -Only adrenaline -Only norepinephrine -Mixed			0.51 [0.12-2.23], 0.331 2.39 [0.95-6.04], 0.069 0.51 [0.19-1.36], 0.165
Urine metanephrine (SD)	6.7± 21.72	6.6± 15.20	1.00* [0.97-1.03], 0.908
Urine normetanephrine (SD)	32.8± 66.7	5.5± 8.66	1.02* [1.01-1.03], <0.0001†
Urine epinephrine (SD)	0.6 ± 0.37	4.9± 8.84	0.37* [0.16-0.89], <0.0001†
Urine norepinephrine (SD)	6.5± 9.15	4.7 ± 12.66	1.00* [0.98-1.04], 0.606
Plasmatic metanephrine (SD)	3.2 ± 5.19	4.9 ± 6.50	0.95* [0.83-1.08], 0.381
Plasmatic normetanephrine (SD)	7.7 ± 9.22	4.5 ± 5.60	1.04* [0.98-1.11], 0.282
Tumour size (mm)	65.7 ± 7.85	46.3 ± 31.27	1.10 per each 10 mm [1.04-1.17], 0.018†
Tumour >40 mm	69.6% (n=16/23)	49.3% (n=132/268)	2.34 [0.96-5.70], 0.050
Bilateral tumors	0%	5.0% (n=14)	Not calculable
Sympathetic paraganglioma	33.3% (n=8)	2.5% (n=7)	11.9 [5.02-28.08], <0.0001
Open adrenalectomy	37.5% (n=9)	11.1% (n=31)	3.62 [1.58-8.29], <0.003
PASS score [n=70]	3.8 ± 3.25	2.9 ± 2.50	1.27 per each increase in unit [0.91-1.77], 0.158

PASS, Pheochromocytoma of the Adrenal gland Scaled Score; PPGL, pheochromocytomas and sympathetic paragangliomas; SD, standard deviations; *refers to each increase in unit; † refers to statistically significant results; **for variables with missing data, the number of patients with available data is described in brackets.

The mean time free of recurrence was of 5.2 ± 3.47 years in patients with pathogenic variants in *SDHB* and of 3.1 ± 2.73 years in those harboring *SDHB* pathogenic variants (P=0.024).

## 4 Discussion

Recurrence of PPGL after its resection occurred in 8% of our patients after a mean follow-up of 5 years, with a rate of local recurrence of 2.3% and of metastatic disease of 7%. Nevertheless, the reported rates of recurrence (local and metastatic together) widely range between 6 to 22% in other series (6, 8, 22, 23). Specifically, local recurrence is reported in 0% of a series of 135 pheochromocytomas after a median follow-up time of 10 years (24) and in 6% of pheochromocytomas after a median follow-up of 4 years in a series of 52 cases (6). Neumann series of pediatric patients described local recurrence in 50% of the patients after 30 years of follow-up (25). An intermediate rate has been described in Amar L series that included 176 cases of PPGL at risk of recurrence, reporting local recurrence of 8% but after a larger follow-up period, of 9 years (8). In this same series, metastases occurred in 15 out of the 176 PPGLs (8.5%). Nevertheless, only 3% of the patients of the O’Dwyer PJ study developed metastasis after a median follow-up of 10 years (24) and as high as 24% in patients with PGLs in other different study (26). The differences described across these studies may be explained by several factors, including differences in the duration of the follow-up period (for example of 30 years in Neumann series (25) and 4 years in Johnston PC study (6)), the inclusion criteria (for example, in series that include extraadrenal tumors the rate of recurrence is higher (8), also in pediatric patients, who have a higher rate of hereditary disease, e.g of 80% of the patients in Neumann series were hereditary), the definitions used for recurrent disease and the diagnosis method employed.

Importantly, despite that in our cohort the median time from the diagnosis to the recurrence was only of 11.2 months, the cases of recurrent disease distributed uniformly during the follow-up period, and they can occur even after 10 years of follow-up. In this line, in the Johnston CP series, all cases or local recurrence occurred at least after 8 years of the surgery for the primary pheochromocytoma (6). In contrast, a meta-analysis of 13 studies, described a lower rate of local recurrence (3%) and a larger mean time to local recurrence (4 years, ranging from 0.5 to 12 years) (27). In addition, in the recent study of Pamporaki (28), 29% of all patients with recurrent sporadic PPGL, were diagnosed with recurrence at least 10 years after primary tumor diagnosis. For these reasons and given the limitations to predict the development of recurrent disease, the current recommendation is an extended long-term follow-up for all patients with PPGLs (9).

We identified as the strongest predictor of recurrence the presence of a pathogenic variant in the *SDHB* gene (HR 13.3, 95% CI 4.20-41.92). According with our results, a recent study found that *SDHB* immunohistochemistry is an important predictive factor of recurrence for PPGL, specifically, in a prospective evaluation they found that 18.8% (3/16) of participants in the *SDHB* (-) group had progressive tumors compared with 3.6% (7/197) in the *SDHB* (+) group (RR: 5.28, 95% CI: 1.51-18.47) (23). In the same line, in a study of 44 malignant PPGLs and 113 benign PPGLs, all 11 patients with germline *SDHB* mutations had malignant disease (29). In agreement with these information, it has been reported that the prevalence of recurrence among patients with sporadic PPGL (14.7%) is lower (P<0.001) than for patients with pathogenic variants that activate pseudohypoxia pathways (47.5%), but similar to those with variants that activate kinase pathways (14.9%) (28). *SDHB* is also a predictive factor of survival in patients with metastatic PPGL, the relative risk of mortality (SDHB mutated vs non-mutated) is of 2.7; [95% confidence interval 1.2 to 6.4] in the Plouin PF study (26). In agreement with these results, we observed that recurrent disease occurred earlier in *SDHB* mutated patients in comparison with those without the mutation, suggesting a more aggressive disease in the former. The same observation was reported in the Plouin PF study: the median time from the diagnosis of primary tumor to the documentation of a first metastasis was 4 months in patients with *SDHB* mutations and 20 months in patients without (26). Thus, when a *SDHB* pathogenic variant is detected, we recommend performing a strict biochemical and radiological follow-up with a special attention for any data that may suggest recurrence development, including an increase in 3-methoxytyramine levels or indeterminate radiological findings. Moreover, the type of *SDHB* pathogenic variants may also be useful to indicate the potential risk for metastatic disease development (30). We also observed that patients with sympathetic PGLs had a higher risk of recurrent disease, but these differences disappeared after adjusting by *SDHB* mutation. This fact may be explained because the clinical behavior of the PGL vary depending on if the case is sporadic or if genetic. Moreover, the clinical behavior of the PGL depends on the underlying pathogenic variant; for example for *SDHD* mutations the risk of malignancy is estimated to be of 4% whereas for *SDHB* mutation, the risk is of up to 50% (10).

A higher urinary normetanephrine excretion was an independently factor of recurrence (HR 1.02 per each increase in standard deviation above the upper limit of normality). In addition, we observed that those patients who developed recurrence tended to have lower urinary epinephrine excretion than those without recurrence. These results are in line with the recently reported by Pamporaki C. et al. (28). In this study, a noradrenergic/dopaminergic phenotype (HR 2.73; 95% CI, 1.55-4.80), larger size (HR 1.82; 95% CI, 1.11-2.96) and extra-adrenal location (HR 1.79; 95% CI, 1.00-3.19) were independent predictors of recurrence in sporadic PPGL. In accordance with these results, the rate of noradrenergic/dopaminergic tumors in recurrent disease was higher than in non-recurrent PPGLs (67.1% vs 32.8%, P<0.001). Moreover, several years ago a retrospective study of 83 PPGLs described those patients with higher levels of dopamine, norepinephrine and aromatic l-amino acid decarboxylase, as well as lower ratios of epinephrine/epinephrine+norepinephrine, had significantly shorter metastases-free intervals (31). Furthermore Eisenhofer et al. (28) conducted a study on 365 PPGLs patients and demonstrated higher norepinephrine, normetanephrine, and 3-methoxytyramine levels in metastatic PPGLs. They found that 3-methoxytyramine was the most accurate biomarker of metastatic disease because plasma methoxytyramine was 4.7 times higher in patients with metastases compared to patients without metastases, and high plasma methoxytyramine was associated with SDHB mutations and extra-adrenal disease, both recognized risk factors of metastatic disease.

In our cohort, large tumor size increased the risk of recurrence during follow-up (HR 1.01 per each increase in mm, 95%CI 1.00-1.02), this finding agrees with several previous studies (16, 32, 33). Tumor size of 5-5.5 cm has been proposed as the most sensitive threshold to differentiate patients with low and high risk of developing recurrent disease (11, 32), additionally, tumor size < 4 cm has been linked to a lower risk of metastatic disease after 7.3 years according to the Dhir M. study (29). Moreover, tumor size is a prognosis factor for metastatic PPGL (16, 32). In this line, a series of 152 patients with pheochromocytoma, including 5 with metastasis at the time of the initial surgical excision and 12 who developed metastasis during follow-up, described a greater overall 5-year progression free survival rate in patients with smaller tumors (≤5.5 vs. >5.5 cm; 90.6% vs. 81.2%, p=0.025) (32). Similar results were reported by Ayala-Ramirez M et al. (16, 34), in which tumor size was significantly associated with worse overall survival (HR = 1.08; 95% CI = 1.04–1.13; P = 0.0003) while adjusting for age, gender, and tumor location.

We also found that patients who underwent surgery using an open approach presented a risk of recurrence three times higher than those who were operated laparoscopically. The open approach is preferred when there is a clinical suspicion or concern for an invasive malignant pheochromocytoma. In addition, larger tumors are at a higher risk for tumor rupture, which may lead to pheochromocytomatosis. However, we observed that even after adjusting by tumor size, open approach was associated with a higher risk of recurrence. Nevertheless, when open approach was adjusted by SDHB mutational status, these differences disappeared. An open adrenalectomy approach may be justified in patients with SDHB mutations due to the higher rate of metastatic disease in this group (35).

Histopathological information may also be useful to predict the development of metastatic disease. However, we did not find differences in the score obtained in the Pheochromocytoma of the Adrenal gland Scaled Score (PASS) system between patients who developed recurrence and those who remained without recurrence. Specifically the Grading System for Adrenal Pheochromocytoma and Paraganglioma (GAPP) criteria include six variables: histological pattern, cellularity, comedo-type necrosis, capsular/vascular invasion, Ki67 labelling index and catecholamine type (36) or the PASS score (37) based on 12 histologic features such as diffuse growth, high cellularity, cellular monotony, tumor cell spindling, mitotic figures [3/10 HPF, atypical mitoses, profound nuclear pleomorphism and nuclear hyperchromasia. However, several of the features are associated with inter- and intra-observer variability and histologic features have not been shown to reliably predict malignancy in PPGLs (38). Other authors have proposed the application of compositive predictive scores combining different clinical and histological variables for the prediction of the progression-free survival (PFS) and metastatic behavior of PPGLs. In this regard, Pierre et al. (39) proposed a new prognostic score called COPPS that combines the variables tumor size, necrosis, vascular invasion and the losses of PS100 and *SDHB* immunostaining to predict the risk of metastasis. Cho et al. (40) proposed another integrated risk score for recurrence prediction called ASES-score, using the variables age ≤35 years, tumor size ≥ 6.0 cm, extra-adrenal localization, and norepinephrine-secretory type. The negative predictive value of this system was 96.5% for a cut-off point of 2. Other similar models have been reported by other authors (41).

We are aware that this is a retrospective study with associated limitations, including cases selection and referral bias. In addition, we have analyzed the predictive value of histological characteristics for development of recurrent disease in few patients, so the power of this analysis to detect differences it is low. Nevertheless, we included a large sample size and a long mean follow-up period, which is one of the main strengths of the study. Furthermore, we have evaluated the influence of several characteristics in the risk of development both metastatic and local recurrent disease, including clinical, genetic, hormonal and radiological data.

## 5 Conclusion

Disease recurrence of PPGLs occurs more frequently in patients with mutations in *SDHB*, with larger tumors and higher levels of urinary normetanephrine. PPGL recurrence may occur after the initial PPGL diagnosis is performed, thus, we recommend genetic testing in all patients with PPGL and a strict follow-up, especially in those patients with a higher risk of recurrent disease.

## Data availability statement

The raw data supporting the conclusions of this article will be made available by the authors, without undue reservation.

## Ethics statement

All procedures performed in the participants of the study were in accordance with the ethical standards of the institutional research committee and with the 1964 Helsinki declaration and its later amendments or comparable ethical standards. The study has been approved by the Ethical Committee of the Hospital Universitario La Princesa and Hospital Universitario Ramón y Cajal. This retrospective multicenter study was approved, and waiver of informed consent was granted by the Hospital Universitario Ramón y Cajal Ethics’ Committee.

## Author contributions

MA-C: Conceptualization, Investigation, Writing – original draft, Writing – review & editing. IG: Writing – review & editing. CM: Writing – review & editing. FH: Writing – review & editing. MM: Writing – review & editing. AV: Writing – review & editing. CB: Writing – review & editing. PN: Writing – review & editing. ML: Writing – review & editing. CL: Writing – review & editing. LM-M: Writing – review & editing. MC: Writing – review & editing. PR: Writing – review & editing. RB: Writing – review & editing. MR: Writing – review & editing. MT: Writing – review & editing. NV: Writing – review & editing. PG: Writing – review & editing. CR: Writing – review & editing. TM: Writing – review & editing. CA: Writing – review & editing. RG: Writing – review & editing. VB-T: Methodology, Writing - review & editing. AH-M: Writing – review & editing. MC: Writing – review & editing.
